# Investigation of the action of poly(ADP-ribose)-synthesising enzymes on NAD^+^ analogues

**DOI:** 10.3762/bjoc.13.49

**Published:** 2017-03-10

**Authors:** Sarah Wallrodt, Edward L Simpson, Andreas Marx

**Affiliations:** 1 Department of Chemistry, University of Konstanz, Universitätsstraße 10, 78457 Konstanz, Germany

**Keywords:** ARTD, click chemistry, NAD^+^, poly(ADP-ribose), posttranslational modification

## Abstract

ADP-ribosyl transferases with diphtheria toxin homology (ARTDs) catalyse the covalent addition of ADP-ribose onto different acceptors forming mono- or poly(ADP-ribos)ylated proteins. Out of the 18 members identified, only four are known to synthesise the complex poly(ADP-ribose) biopolymer. The investigation of this posttranslational modification is important due to its involvement in cancer and other diseases. Lately, metabolic labelling approaches comprising different reporter-modified NAD^+^ building blocks have stimulated and enriched proteomic studies and imaging applications of ADP-ribosylation processes. Herein, we compare the substrate scope and applicability of different NAD^+^ analogues for the investigation of the polymer-synthesising enzymes ARTD1, ARTD2, ARTD5 and ARTD6. By varying the site and size of the NAD^+^ modification, suitable probes were identified for each enzyme. This report provides guidelines for choosing analogues for studying poly(ADP-ribose)-synthesising enzymes.

## Introduction

**Figure 1:**
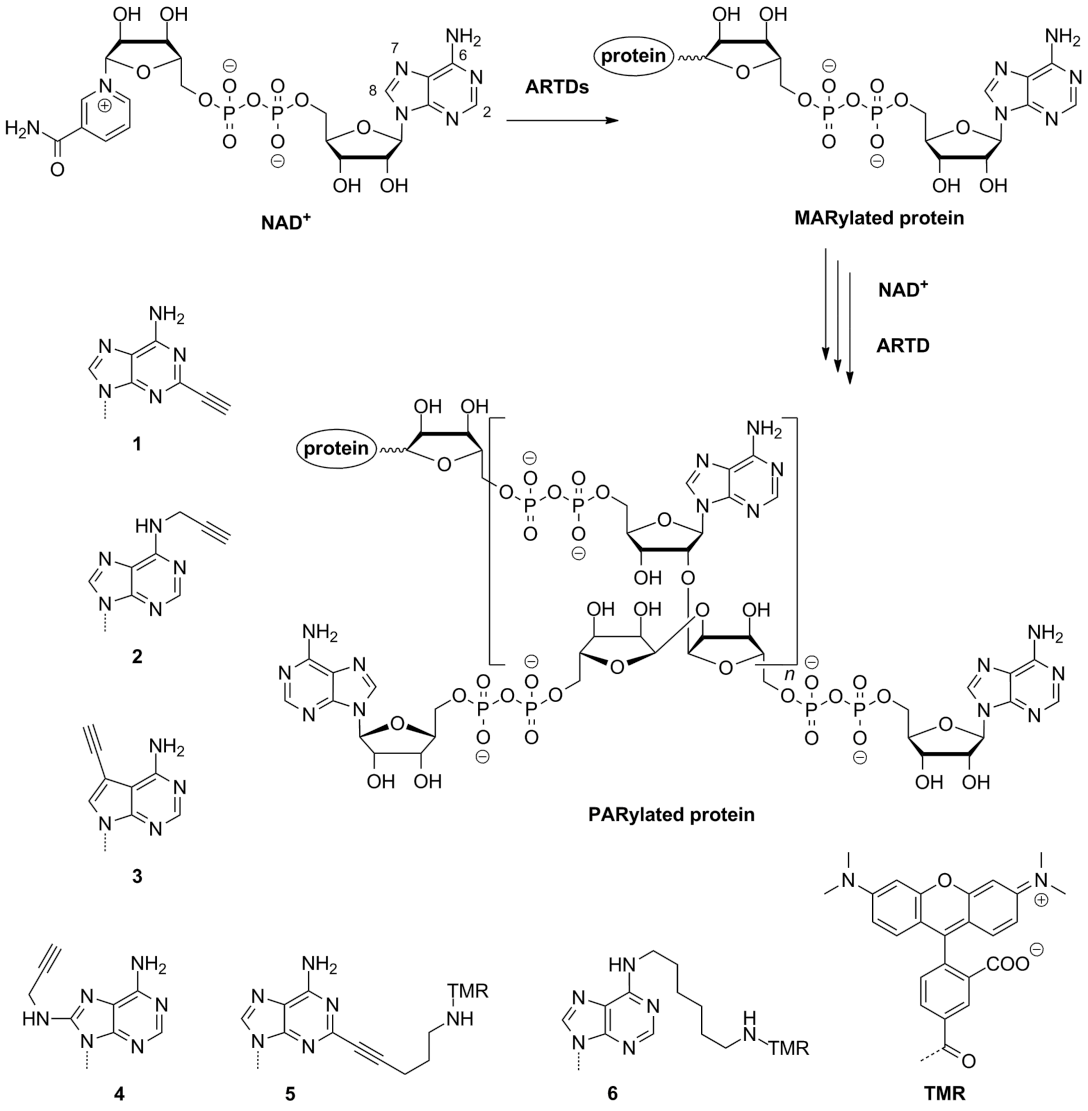
NAD^+^ is used as a substrate by ARTDs to form MARylated and PARylated proteins. Depicted are alkyne- and dye-modified NAD^+^ analogues **1**–**6** that are applied in this study.

ADP-ribosyl transferases with diphtheria toxin homology [1] (ARTDs), also termed poly(ADP-ribose) polymerases (PARPs), form an enzyme family of 18 human members [2] that mediate their widespread functions in cellular homeostasis through the catalysis of ADP-ribosylation [3–4]. This posttranslational modification received considerable attention within the last decade [5–6] and has been linked to tumour biology, oxidative stress, inflammatory, and metabolic diseases [7]. Using NAD^+^ as a substrate, ARTDs covalently transfer ADP-riboses onto themselves or different targets forming mono(ADP-ribos)ylated proteins. Some ARTDs are in particular able to elongate these initial units with additional NAD^+^ molecules to build a complex, highly charged biopolymer called poly(ADP-ribose) (PAR, Figure 1). These polymers consist of up to 200 units of ADP-ribose and may branch every 20 to 50 monomers [8–10]. To date, only four ARTD members were found to accomplish the synthesis of PAR, namely the DNA-dependent ARTD1 and ARTD2 as well as the tankyrases ARTD5 and ARTD6 [2–3].

ARTD1 as the founding member is the best investigated enzyme of ARTDs and is considered the main source of cellular PAR [11]. ARTD1 and its closest relative ARTD2 comprise DNA-binding domains and their activity is stimulated by binding to different types of DNA breaks [12]. They fulfil functions in DNA repair, genome maintenance, transcription, and metabolic regulation [11,13]. The tankyrases ARTD5 and ARTD6 also exhibit a unique domain structure consisting of multiple ankyrin repeats mediating protein–protein interactions [13]. Tankyrases are involved in telomere homeostasis, Wnt/β-catenin signalling, glucose metabolism, and cell cycle progression [14].

Remarkable efforts have been undertaken to develop tools and assays for studying PARylation on a molecular level and to understand the complex processes and interactions of the involved ARTDs. Recently, the employment of NAD^+^ analogues resulted in the development of powerful applications for the determination and visualisation of ARTD activity [15–18], the identification of PARylation sites and targets [15,19–20] and the real-time imaging [21] of PARylation processes.

In this report, we systematically compare the substrate scope of the four poly(ADP-ribose)-synthesising enzymes ARTD1, ARTD2, ARTD5 and ARTD6. For this purpose, we tested reporter-modified NAD^+^ analogues **1**–**6** (Figure 1) that were previously applied in ARTD1 catalysed ADP-ribosylation [15,17,21]. By investigating them in biochemical assays, we identified sites and sizes of modifications for each enzyme that are well-accepted and competitively used in the presence of natural substrate. In this way, new insights of the enzyme’s substrate scope and the applicability of NAD^+^ analogues are gained and should thus guide future experiments.

## Results and Discussion

### Alkyne-modified NAD^+^ analogues

First, the position of the reporter group is systematically varied by introducing small, terminal alkyne functionalities at common sites of the adenine base. Upon successful incorporation into PAR, these alkynes serve as handles for copper(I) catalysed azide–alkyne click reaction (CuAAC) [22] with fluorescent dyes. Terminal alkynes are the smallest possible reporter group that allows the selective labelling of poly(ADP-ribose) [17]. As reported, the synthesis of alkyne-modified derivatives **1**–**4** was previously [16–17,23] accomplished by preparing the respective alkyne-modified nucleosides from common precursors and turning them into their corresponding NAD^+^ analogues in a two-step procedure (Supporting Information File 1, Scheme S1).

**Figure 2:**
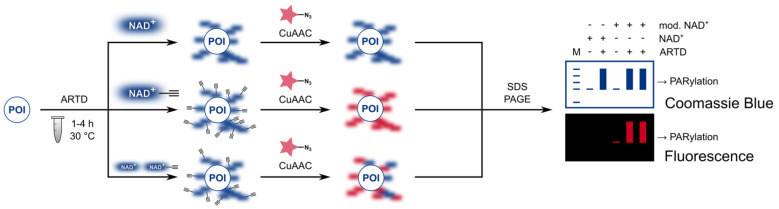
Workflow of the ADP-ribosylation assay. The protein of interest (POI) is ADP-ribosylated by the respective ARTD and by NAD^+^, NAD^+^ analogue or a 1:1 mixture. Then, copper(I)-catalysed azide–alkyne click reaction (CuAAC) is performed and mixture is resolved by SDS PAGE.

Next, NAD^+^ substrate properties were investigated in ADP-ribosylation assays with histone H1.2 as acceptor and in ARTD automodification. For a better comparison, the assay conditions for ARTD2, ARTD5 and ARTD6 were chosen to be similar and were derived from previously established ARTD1 catalysed ADP-ribosylation [21]. Incubation of NAD^+^ or NAD^+^ analogues with ARTD enzyme in reaction buffer and with or without histone H1.2 as additional acceptor protein were performed at 30 °C to decrease the reported NADase activity of tankyrases [15]. Reaction times were elongated to 1 h, 4 h and 2 h, respectively, to achieve noticeable PAR formation. Moreover, no DNA was added to the tankyrase reactions. Of note, ARTD2 was found to be not activated by short, octameric DNA such as applied in case of ARTD1 and thus activated calf thymus DNA was added to enable ARTD2 catalysed PAR production [24]. After the times indicated, copper-catalysed click conjugations to a fluorophore-containing azide were performed and the reactions were analysed by SDS PAGE. Then, fluorescent signals were detected and compared to the Coomassie Blue stained gels (Figure 2). Each analogue was additionally tested in a 1:1 mixture with natural NAD^+^ to explore their competitiveness against natural substrate and all gels contain controls without enzyme. A positive PARylation reaction is indicated by heterogeneous, polymer-modified proteins and/or the reduction of the ARTD band due to automodification. If analogues are successfully incorporated, the polymer chains can additionally be detected in the fluorescence read-out.

**Figure 3:**
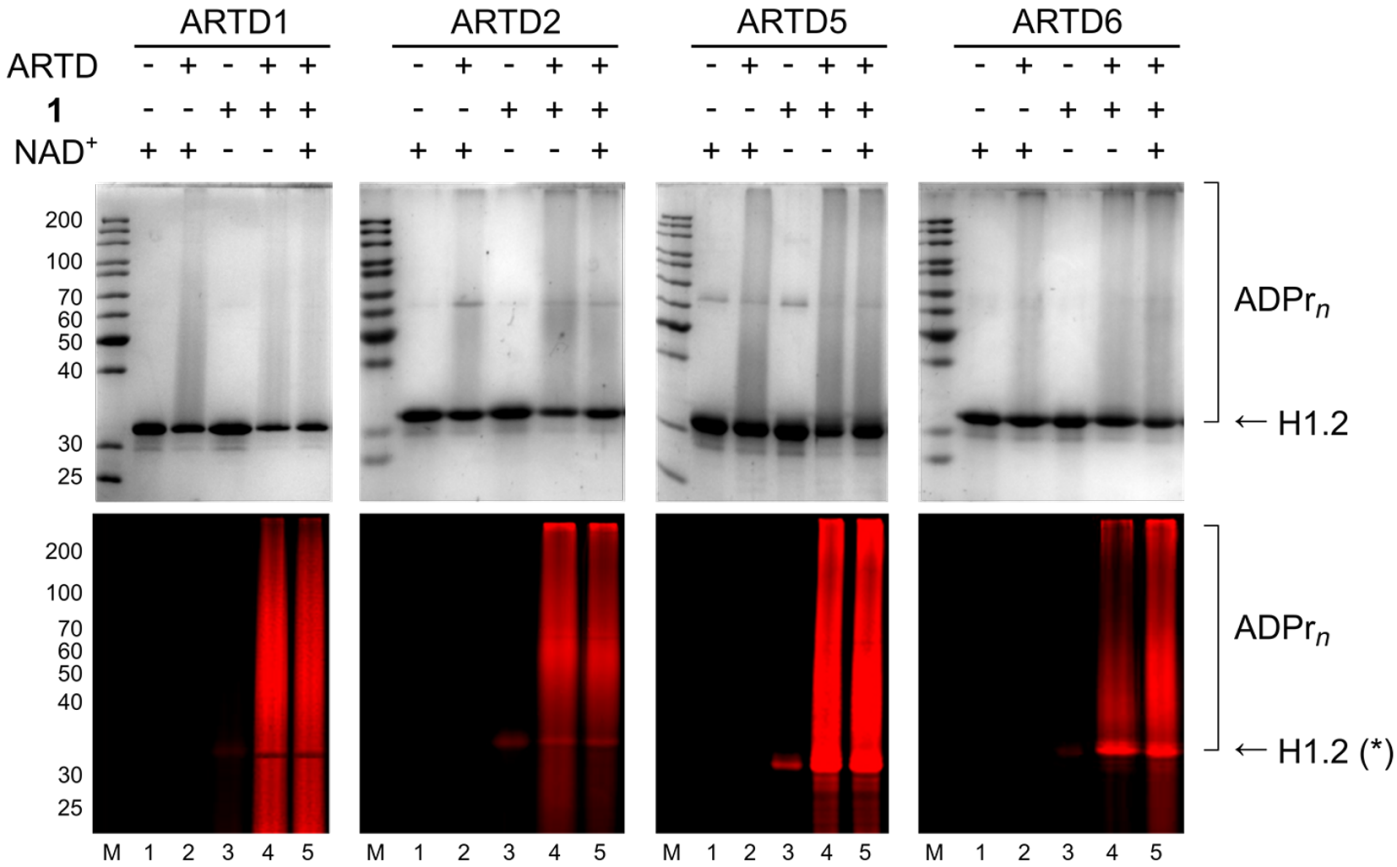
SDS PAGE analysis of ADP-ribosylation of histone H1.2 with ARTD1, ARTD2, ARTD5 and ARTD6 using NAD^+^ analogue **1**. Upper panel shows Coomassie Blue staining; lower panel shows TMR fluorescence. Experimental details are provided in Supporting Information File 1. *Unspecific staining of H1.2 in lanes 3 results from non-catalytic bond formation of NAD^+^ analogues with the protein.

For a better comparison, ARTD1-based ADP-ribosylation assays were also performed, because all four analogues have never been tested in parallel before. The outcome of these experiments is summarised in Table 1. For illustration, Figure 3 shows the processing of derivative **1** by all the four ARTDs tested. Of note, it was previously reported [21] that the incubation of proteins with NAD^+^ analogues may result in non-enzymatic Schiff base formation of ADP-riboses with lysine residues [25] and can be detected by some minor staining of the involved proteins, which is also visible in some of the investigated reactions.

**Table 1:** Acceptance of alkyne-modified NAD^+^ analogues **1**–**4** by different ARTDs without or with competition of natural substrate.^a^ 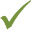 = analogue is well processed, 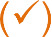 = analogue is processed with lower efficiency, 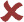 = analogue is not processed.

NAD^+^ analogue	Nat. NAD^+^	ARTD1	ARTD2	ARTD5	ARTD6

**1**	–	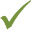	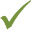	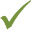	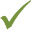
	1:1	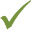	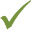	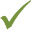	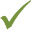
**2**	–	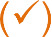	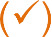	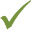	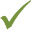
	1:1	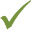	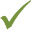	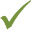	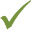
**3**	–	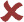	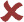	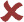	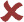
	1:1	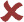	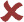	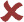	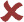
**4**	–	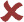	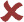	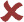	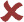
	1:1	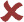	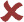	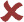	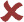

^a^All gels are depicted in Supporting Information File 1, Figure S1 and Figure S2.

As expected from the close structural similarity between ARTD1 and ARTD2 (panel a and b), both enzymes behave similarly in histone ADP-ribosylation (Supporting Information File 1, Figure S1) and in auto(ADP-ribos)ylation (Figure S2). As known from previous work [15,17], ARTD1 was not able to process 7- and 8-modified NADs **3** and **4** and so does ARTD2 (Supporting Information File 1, Figure S1, lanes 9 to 14 and Figure S2, lanes 7 to 10). In both assays, only small amounts of modified PAR was formed with the 6-modified derivative **2** and in the absence of natural NAD^+^ (Supporting Information File 1, Figure S1, lane 7 and Figure S2, lane 5), when compared in parallel with 2-modified analogue **1**. However, a strong signal is detected in a mixture containing NAD^+^ (Figure S1, lane 8 and Figure S2, lane 6). Application of compound **1** results in the strongest signal and is competitive towards natural substrate (Figure S1, lanes 4 to 5 and Figure S2, lanes 3 to 4).

Also in ARTD5- and ARTD6-catalysed ADP-ribosylation (panel c and d), analogues **3** and **4** were not used as substrates (Supporting Information File 1, Figure S1, lanes 9 to 10 and Figure S2, lanes 7 to 10). In contrast, compounds **1** and **2** were both used by both enzymes for PAR formation, even in the absence of natural NAD^+^. In case of ARTD5, derivative **1** seems to be slightly better processed than **2** in histone ADP-ribosylation, whereas in case of ARTD6 both are used as substrates in both assays with similar efficiencies.

### Dye-modified NAD^+^ analogues

**Figure 4:**
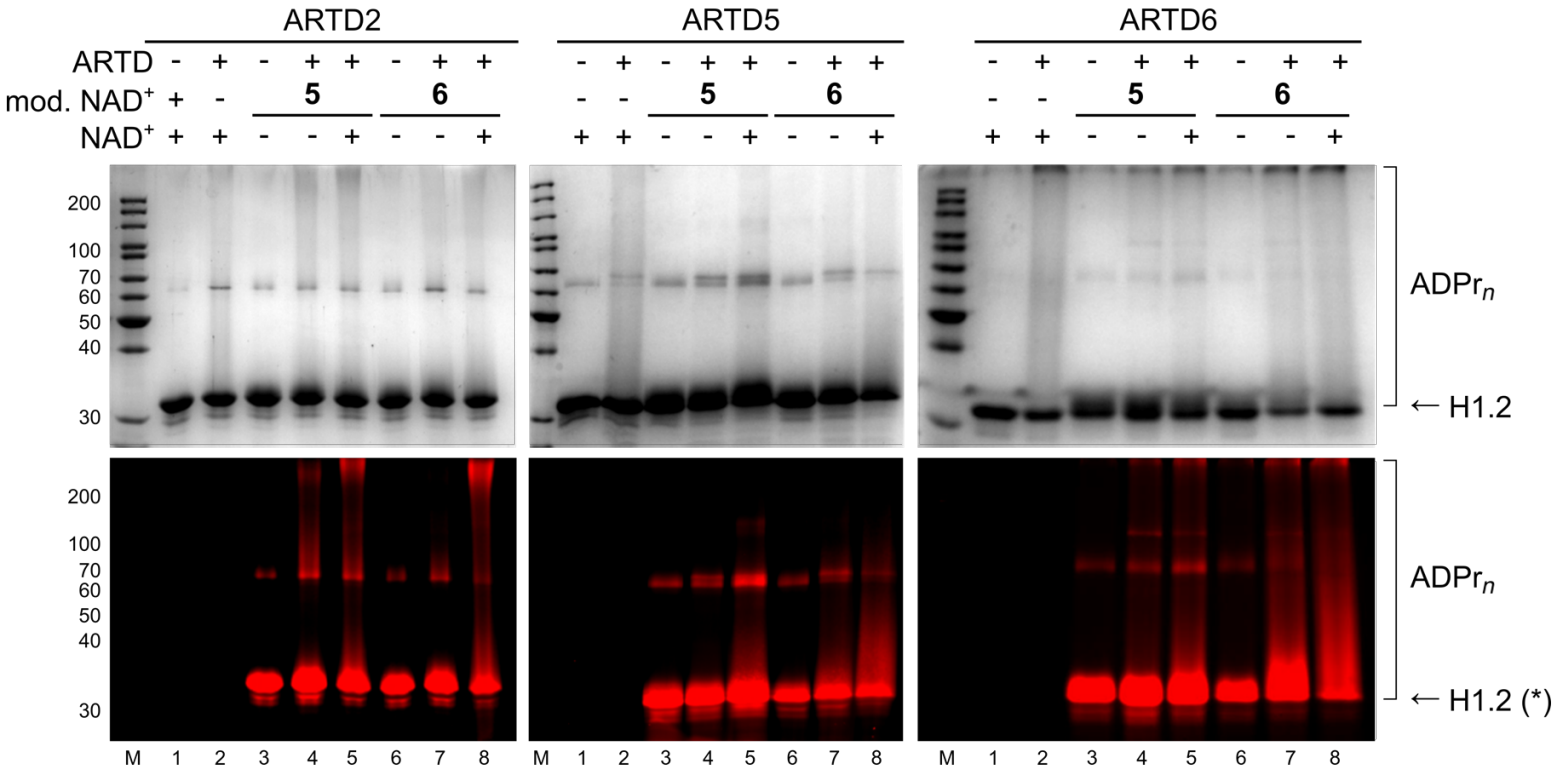
SDS PAGE analysis of ADP-ribosylation of histone H1.2 with ARTD2, ARTD5 and ARTD 6 using NAD^+^ analogues **5** and **6**. Upper panel shows Coomassie Blue staining; lower panel shows TMR fluorescence. Experimental details are provided in Supporting Information File 1. *High unspecific staining of H1.2 in lanes 3 and 6 results from non-catalytic bond formation of NAD^+^ analogues with the protein.

Because the alkyne-tag induces only small alterations to the NAD^+^ scaffold, we also investigated how these enzymes would act on bulkier substitutions. For this purpose, we selected bulky, dye-modified NAD^+^ analogues **5** and **6**, which were previously prepared by our group [21], in order to have a direct, fluorescent read-out. The outcome is summarised in Table 2 and the SDS PAGE gels obtained are depicted in Figure 4 and Supporting Information File 1, Figures S3 and S4.

**Table 2:** Acceptance of dye-modified NAD^+^ analogues **5** and **6** by different ARTDs without or with competition of natural substrate.^a^ 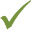 = analogue is well processed, 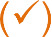 = analogue is processed with lower efficiency, 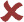 = analogue is not processed.

NAD^+^ analogue	Nat. NAD^+^	ARTD1	ARTD2	ARTD5	ARTD6

**5**	–	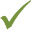	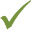	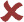	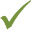
	1:1	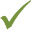	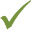	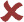	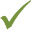
**6**	–	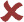	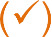 ^b^ 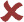	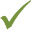 ^c^ 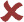	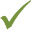
	1:1	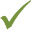	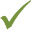	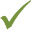 ^c^ 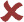	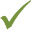

^a^All gels are depicted in Supporting Information File 1, Figure S3 and Figure S4. ^b^**6** is accepted in H1.2 ADP-ribosylation with little efficiency, but not in automodification. ^c^Analogues are not accepted in automodification.

As shown in Figure 4 and Supporting Information File 1, Figure S4b, ARTD2 processes analogue **5** in a competitive manner and fluorescent and Coomassie Blue stained polymer chains are formed in the absence and the presence of natural substrate (Figure 4, lanes 4 to 5 and Figure S4b, lanes 3 to 4). Unlike ARTD1 (Supporting Information File 1, Figure S3a), little fluorescent signal is obtained with compound **6** in ARTD2 catalysed histone PARylation in the absence of natural NAD^+^ (Figure 4, lane 7) and in ARTD2 automodification (Figure S4b, lane 5).

ARTD5 showed decreased incorporation of the larger substituted analogues **5** and **6**. During automodifcation, both compounds failed to form detectable, fluorescent PAR chains (Figure 4 and Supporting Information File 1, Figure S4c, lanes 3 to 6). In general, it can be concluded that ARTD5 showed less activity in automodification compared to the other ARTDs [26]. Nevertheless, analogue **6** was somewhat processed using the histone-based assay as seen by fluorescent and Coomassie-blue-stained polymers in the absence of natural substrate and increased polymer in the presence of natural NAD^+^ (Figure 4, lanes 7 to 8). The fluorescence observed in the presence of **5** is similar to the background signal indicating poor processing of **5** (Figure 4, lanes 4 to 5).

In case of ARTD6, both analogues were used for the ADP-ribosylation of histone (Figure 4, lanes 3 to 8) and in automodification (Supporting Information File 1, Figure S4d, lanes 3 to 6) with similar efficiency.

## Conclusion

In this paper, we investigated the scope of PAR synthesising enzymes, namely ARTD1, ARTD2, ARTD5 or ARTD6 for using modified NAD^+^ analogues. It was found that NAD^+^ analogues **1** and **2** modified with alkyne groups in adenine position 2 and 6 are used by all these enzymes to a certain extent, whereas the employed substitutions in adenine at position 7 and 8 completely abrogated the processing towards PAR. The DNA-dependent ARTDs ARTD1 and ARTD2 can process 2-modified analogues best as also sterically demanding compounds such as dye-modified **5** are processed. Thus, 2-modified analogues are the best choice for the study of these enzymes. On the other hand, 6-modified derivatives should be chosen for the study of the tankyrases ARTD5 and ARTD6. When bulky substitutions are added on the NAD^+^ scaffold, tankyrases tolerate better 6-modifed analogues. Because ARTD5 and ARTD6 exhibit different constraints for metabolising bulky 2-modified analogue **5**, this behaviour could be used to discriminate their activity in a cellular context. By choosing the best NAD^+^ substrate for each enzyme more reliable and valuable insights into PARylation can be achieved and will help to decipher these processes in more detail.

## Supporting Information

File 1 Additional figures, synthesis of compounds and biochemical methods.
