# Supplementary material for: Investigation of the action of poly(ADP-ribose)-synthesising enzymes on NAD+ analogues
Source: Beilstein J Org Chem. 2017 Mar 10;13:495–501. doi: 10.3762/bjoc.13.49 (PMC5355910; doi:10.3762/bjoc.13.49)
Supplement: File 1 — Additional figures, synthesis of compounds and biochemical methods. [file Beilstein_J_Org_Chem-13-495-s001.pdf]

Supporting Information

for

Investigation of the action of poly(ADP-ribose)-synthesising enzymes on NAD$^+$ analogues

Sarah Wallrodt, Edward L. Simpson and Andreas Marx*

Address: Department of Chemistry, University of Konstanz, Universitätsstraße 10, 78457 Konstanz, Germany

Email: Andreas Marx - andreas.marx@uni-konstanz.de

*Corresponding author

Additional figures, synthesis of compounds and biochemical methods

1 Supporting figures S2

2 Synthesis of compounds S6

3 Biochemical methods S7

4 References S11

1 Supporting figures

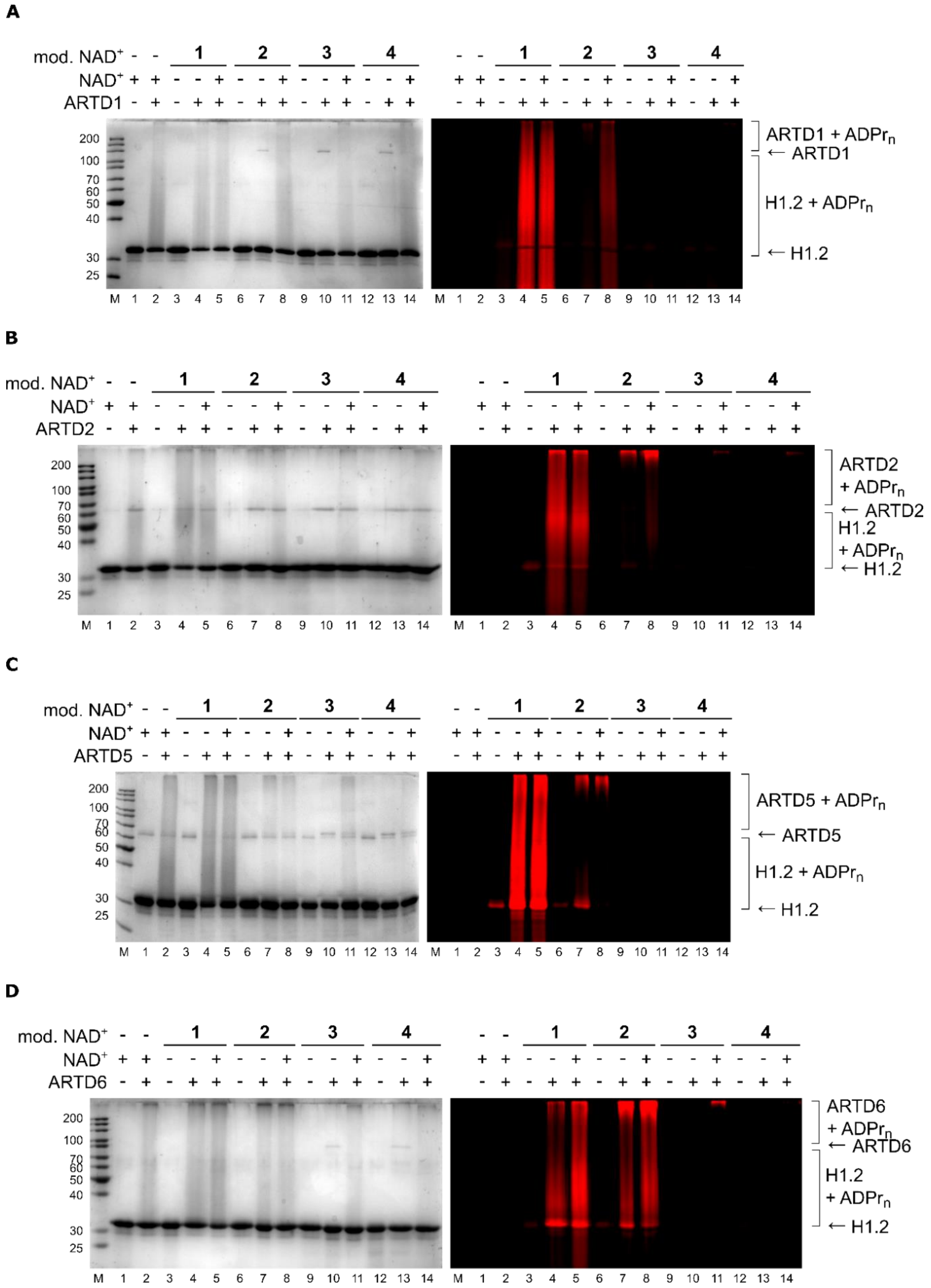

Figure S1: SDS PAGE analyses of ADP-ribosylation of histone H1.2 with ARTD1 (A), ARTD2 (B), ARTD5 (C) and ARTD6 (D) using NAD^+ analogues **1**–**4**. Left panel shows Coomassie Blue staining; right panel shows Cy3 fluorescence. Total concentration of NADs was 1 mM. Controls were performed using either natural substrate (lane 1 and 2) or no enzyme (lane 1, 3, 6, 9 and 12).

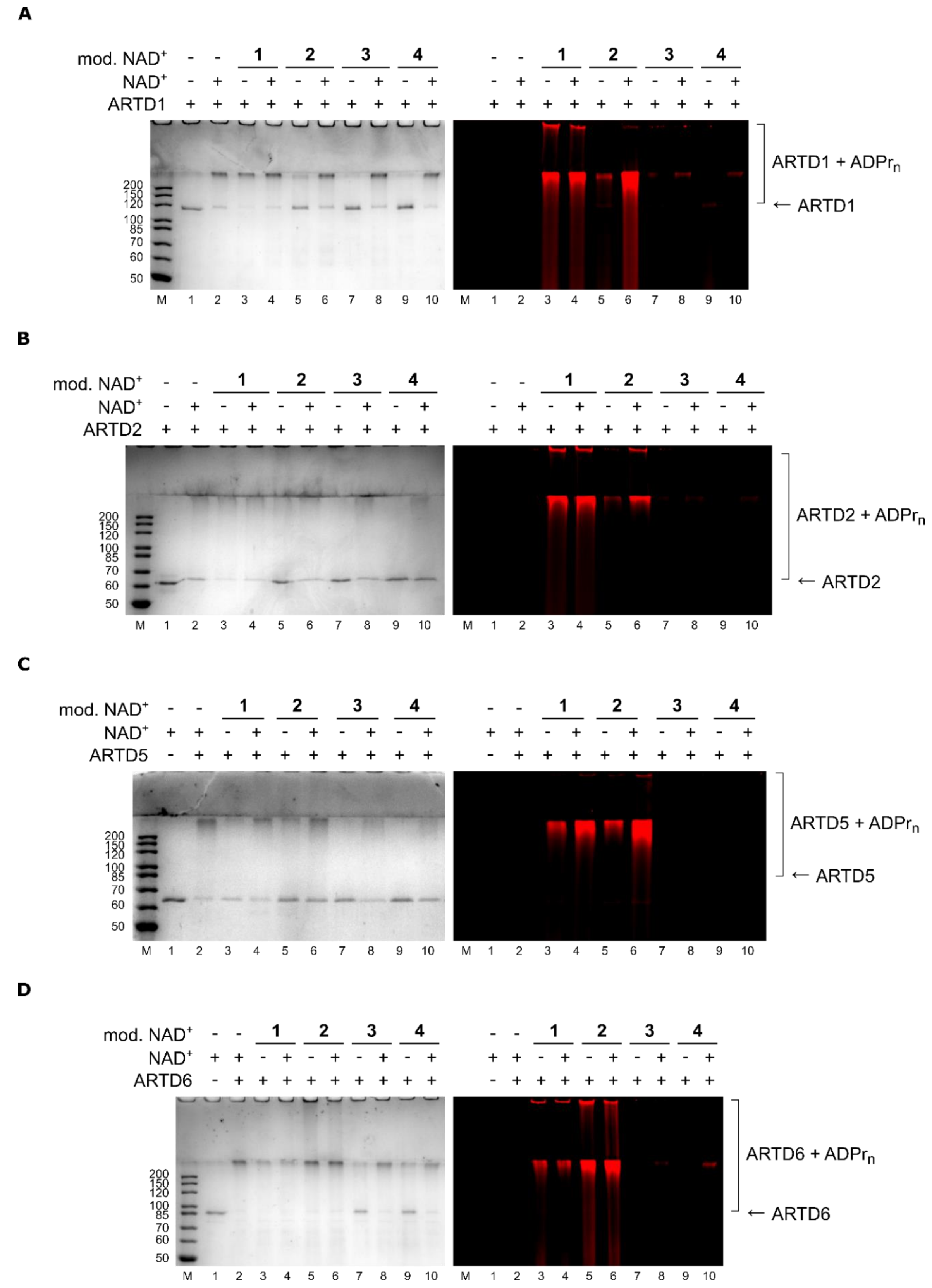

Figure S2: SDS PAGE analyses of auto(ADP-ribos)ylation of ARTD1 (A), ARTD2 (B), ARTD5 (C) and ARTD6 (D) using NAD^+ analogues **1**–**4**. Left panel shows Coomassie Blue staining; right panel shows Cy3 fluorescence. Total concentration of NADs was 1 mM. Controls were performed using either natural substrate (lane 2) or loading the same amount of ARTD1 (lane 1).

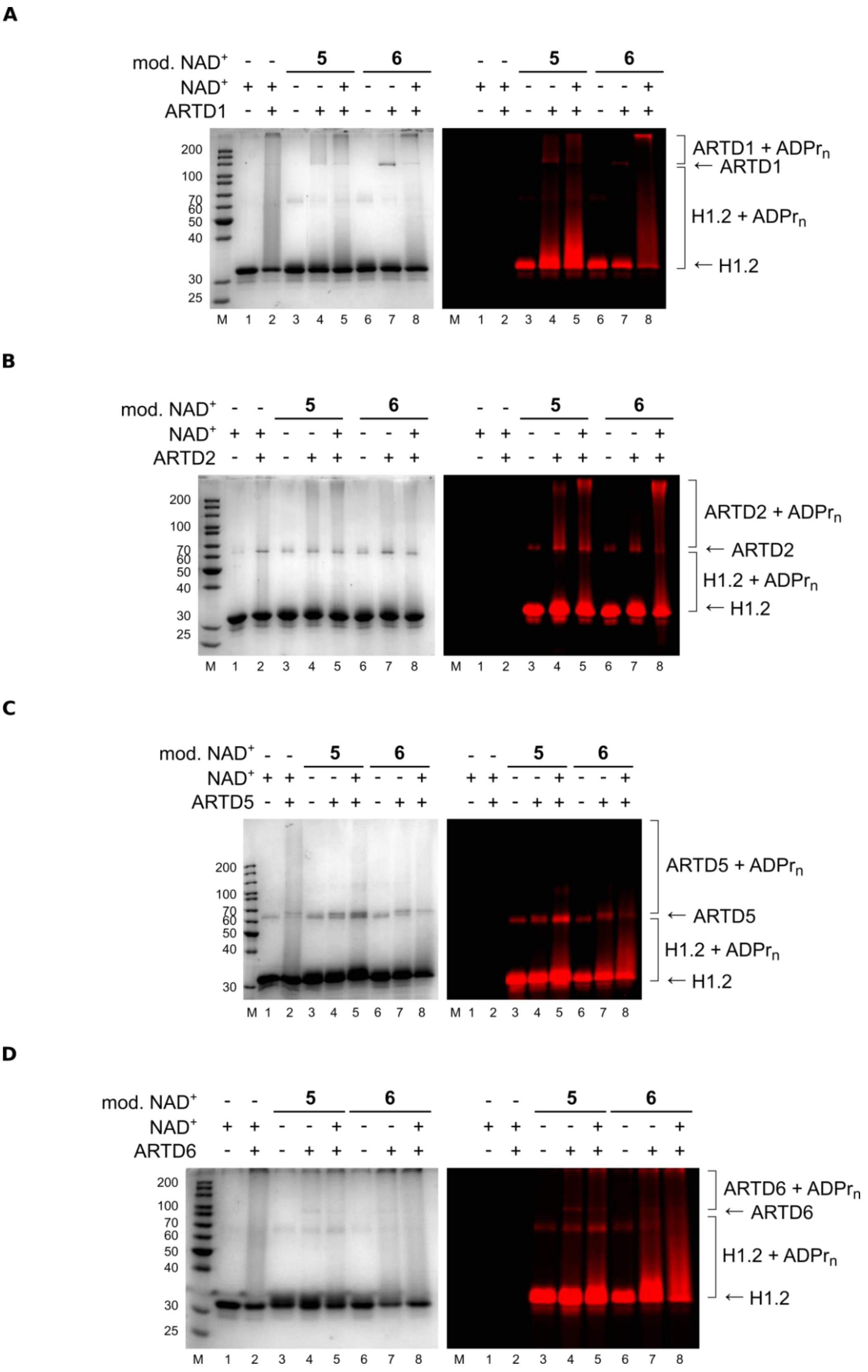

Figure S3: SDS PAGE analyses of ADP-ribosylation of histone H1.2 with ARTD1 (A), ARTD2 (B), ARTD5 (C) and ARTD6 (D) using NAD^+ analogues **5** and **6**. Left panel shows Coomassie Blue staining; right panel shows TMR fluorescence. Total concentration of NADs was 1 mM. Controls were performed using either natural substrate (lane 1 and 2) or no enzyme (lane 1, 3 and 6).

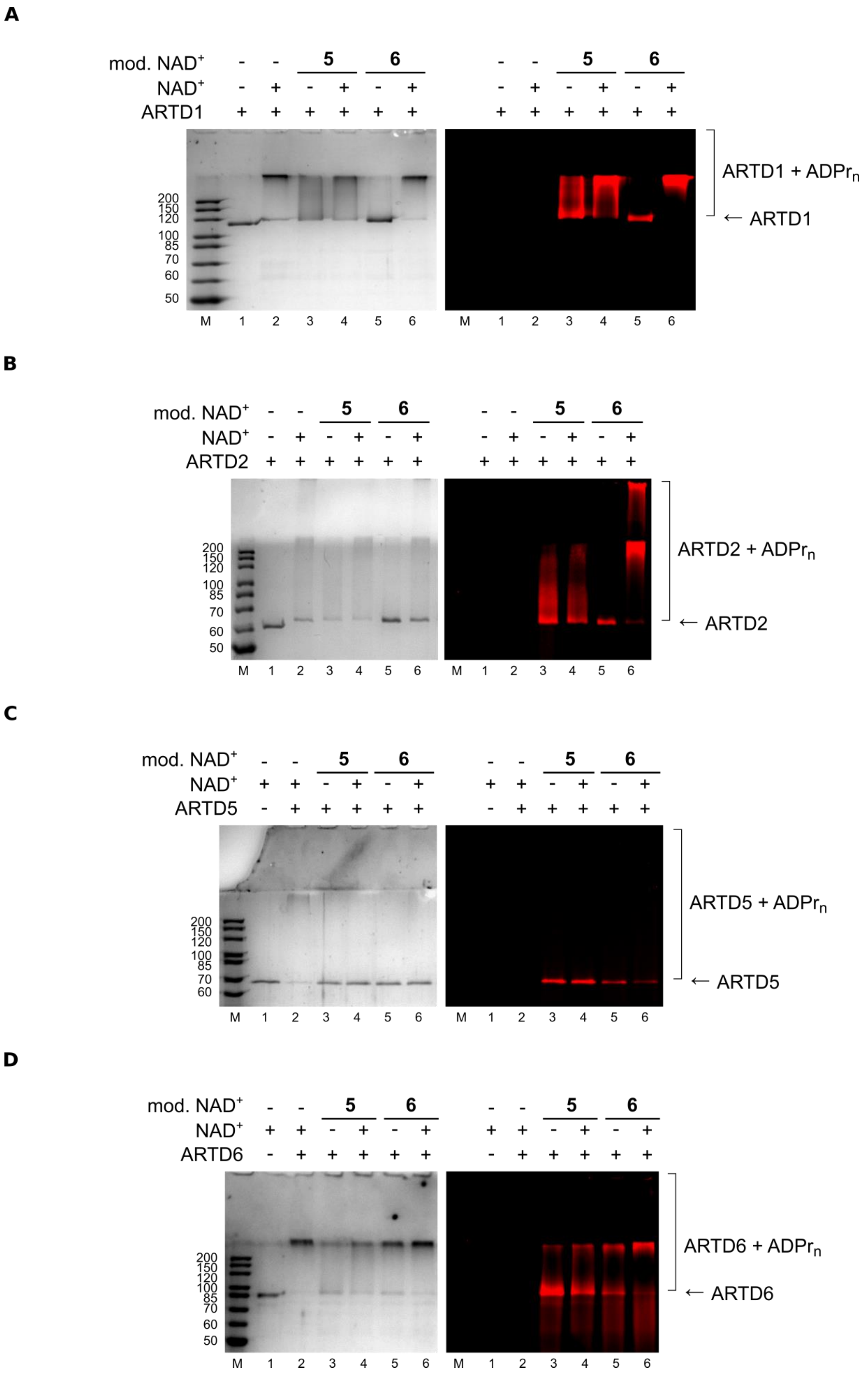

Figure S4: SDS PAGE analyses of auto(ADP-ribos)ylation of ARTD1 (A), ARTD2 (B), ARTD5 (C), and ARTD6 (D) using NAD^+ analogues **5** and **6**. Left panel shows Coomassie Blue staining; right panel shows TMR fluorescence. Total concentration of NADs was 1 mM. Controls were performed using either natural substrate (lane 2) or loading the same amount of ARTD (lane 1).

2 Syntheses of compounds

All syntheses have been previously accomplished and compounds were prepared according to the following scheme. Experimental details and structural characterisation of NAD$^+$ analogues **1–6** and their intermediates are in agreement with literature [1-4].

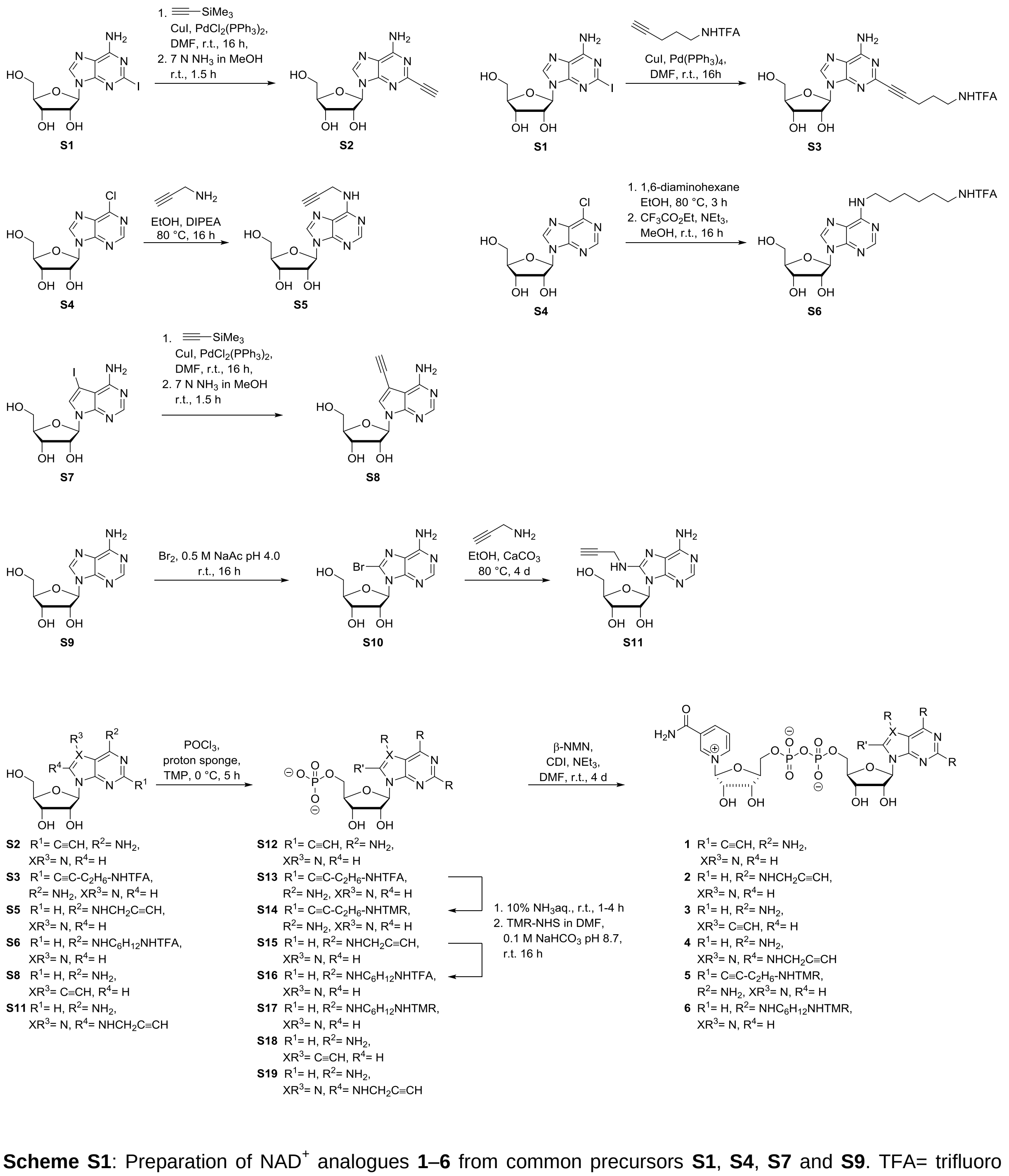

Scheme S1: Preparation of NAD$^+$ analogues **1–6** from common precursors **S1**, **S4**, **S7** and **S9**. TFA= trifluoro acetate, TMR= tetramethylrhodamine.

3 Biochemical methods

3.1 Material

Fluorescent dyes

- Sulfo-Cy3-azide (Cy3-N_3) is commercially available by Jena Bioscience.

Oligonucleotides

- dsDNA: oligonucleotide (5'-GGAATTCC-3') from Invitrogen, was annealed (heated and slowly cooled) prior to use.
- activated Calf thymus DNA was obtained from Sigma Aldrich.

Enzymes and proteins

- human recombinant ARTD1 purchased from Enzo Life Sciences .
- histone H1.2, expressed and purified as described previously[2] Sequence: (M)SETAPAAPAAAPPAEKAPVKKKAAKKAGGTPRKASGPPVSELITKA VAASKERSGVSLAALKKALAAAGYDVEKNNSRIKLGLKSLVSKGTLVQTKGTGASG SFKLNKKAASGEAKPKVKKAGGTKPKKPVGAAKKPKKAAGGATPKKSAKKTPKKA KKPAAATVTKKVAKSPKKAKVAKPKKAAKSAAKAVKPKAAKPKVVKPKKAAPKKK HHHHHH.
- mouse recombinant ARTD2 was obtained from Adipogen Life Science, distributed by Biomol.
- human recombinant ARTD5 (TNKS1) and ARTD6 (TNKS2) were purchased from BPS Bioscience, distributed by Biomol.

Reaction buffer

- PARylation: 100 mM Tris-HCl, pH 7.8; 10 mM $MgCl_2$; 1 mM DTT.

Enzyme storage buffers

Purchased enzymes were diluted to 1 µM solutions, then aliquoted and stored at −80 °C.

- ARTD1: 100 mM Tris-HCl, pH 7.5; 14 mM β-mercaptoethanol, 0.5 mM EDTA, 0.5 mM PMSF, 10% glycerol. Note: PMSF dissolved in dry iPrOH, add immediately and freeze.
- ARTD2: 50 mM Tris-HCl, pH 7.5; 100 mM NaCl, 50 mM imidazol, 0.2% NP-40, 10% glycerol. Do not reuse after defrost.
- ARTD6: 40 mM Tris-HCl, pH 8.0; 110 mM NaCl, 2.2 mM KCl, 0.04% Tween 20, 3 mM DTT, 20% glycerol. Do not reuse after defrost.

ARTD5 is a very sensitive enzyme. It should not be stored diluted. Single use aliquots were prepared are not reused. Before use, defrosted enzyme was diluted in 1× PARylation buffer

- ARTD5: 40 mM Tris-HCl, pH 8.0; 110 mM NaCl, 2.2 mM KCl, 16 mM glutathione, 3 mM DTT, 20% glycerol.

3.2 Biochemical assays

Trans(ADP-ribos)ylation of H1.2 wt with ARTD1 as previously[2-4]

For a typical reaction (5 µL), ARTD1 (150 nM) was added on ice to a mixture containing reaction buffer, dsDNA (5’-GGAATTCC-3’, 13 µM), histone H1.2 (7.2 µM) and NAD^+ analogue (1 mM) and the mixture was incubated for 20 min at 37 °C. Reactions were quenched by the addition of 6× loading dye and denaturation for 5 min at 95 °C. Controls were performed without enzyme and with natural NAD^+ (1 mM). Results were resolved by SDS PAGE (12.5% gels, 35 min at 30 mA). Gels were stained with Coomassie Blue and additional RotiBlue® and recorded with ChemiDoc XRS (BioRad). The image was adjusted in Quantity One 1-D Analysis Software 4.5.1 (BioRad).

Trans(ADP-ribos)ylation of H1.2 wt with ARTD2

For a typical reaction (5 µL), ARTD2 (150 nM) was added on ice to a mixture containing reaction buffer, activated Calf thymus DNA (10 ng/µL), histone H1.2 (7.2 µM) and NAD^+ analogue (1 mM) and the mixture was incubated for 1 h at 30 °C. Reactions were quenched by the addition of 6x loading dye and denaturation for 5 min at 95 °C. Controls were performed without enzyme and with natural NAD^+ (1 mM). Results were processed as indicated above.

Trans(ADP-ribos)ylation of H1.2 wt with ARTD5

ARTD5 single use aliquots were diluted wit 1x PARylation buffer to a concentration of 1 µM. For a typical reaction (5 µL), ARTD5 (150 nM) was added on ice to a mixture containing reaction buffer, histone H1.2 (7.2 µM) and NAD^+ analogue (1 mM) and the mixture was incubated for 4 h at 30 °C. Reactions were quenched by the addition of 6× loading dye and denaturation for 5 min at 95 °C. Controls were performed without enzyme and with natural NAD^+ (1 mM). Results were processed as indicated above.

Trans(ADP-ribos)ylation of H1.2 wt with ARTD6

For a typical reaction (5 µL), ARTD6 (150 nM) was added on ice to a mixture containing reaction buffer, histone H1.2 (7.2 µM) and NAD^+ analogue (1 mM) and the mixture was incubated for 2 h at 30 °C. Reactions were quenched by the addition of 6× loading dye and denaturation for 5 min at 95 °C. Controls were performed without enzyme and with natural NAD^+ (1 mM). Results were processed as indicated above.

Auto(ADP-ribos)ylation of ARTD1 as previously[2,4]

For a typical reaction (5 µL or 10 µL), ARTD1 (150 nM) was added on ice to a mixture containing reaction buffer, dsDNA (13 µM) and NAD^+ analogue (1 mM) and the mixture was

incubated for 20 min at 37 °C. Reactions were quenched by the addition of 6× loading dye and denaturation for 5 min at 95 °C. Controls were performed with natural NAD^+ (1 mM) and by loading same amount of ARTD1. Results were processed as indicated above.

Auto(ADP-ribos)ylation of ARTD2

For a typical reaction (10 µL), ARTD2 (150 nM) was added on ice to a mixture containing reaction buffer, activated Calf thymus DNA (10 ng/µL) and NAD^+ analogue (1 mM) and the mixture was incubated for 1 h at 30 °C. Reactions were quenched by the addition of 6× loading dye and denaturation for 5 min at 95 °C. Controls were performed with natural NAD^+ (1 mM) and by loading same amount of ARTD2. Results were processed as indicated above.

Auto(ADP-ribos)ylation of ARTD5

ARTD5 single use aliquots were diluted wit 1× PARylation buffer to a concentration of 1 µM. For a typical reaction (10 µL), ARTD5 (150 nM) was added on ice to a mixture containing reaction buffer and NAD^+ analogue (1 mM) and the mixture was incubated for 4 h at 30° C. Reactions were quenched by the addition of 6x loading dye and denaturation for 5 min at 95 °C. Controls were performed with natural NAD^+ (1 mM) and by loading same amount of ARTD5. Results were processed as indicated above.

Auto(ADP-ribos)ylation of ARTD6

For a typical reaction (10 µL), ARTD6 (150 nM) was added on ice to a mixture containing reaction buffer and NAD^+ analogue (1 mM) and the mixture was incubated for 2 h at 30 °C. Reactions were quenched by the addition of 6× loading dye and denaturation for 5 min at 95 °C. Controls were performed with natural NAD^+ (1 mM) and by loading same amount of ARTD6. Results were processed as indicated above.

CuAAC Labelling and PAR detection by fluorescence as previously[3]

Trans(ADP-Ribos)ylation or auto(ADP-ribos)ylation assay was performed as described above. Each assay mixture was incubated with Cy3-N_3 (2 mM), THPTA (1 mM) and [Cu(I)$(MeCN)_4BF_4$] (1 mM) for one hour at 0 °C and under argon atmosphere prior to denaturation. The reaction mixtures were subsequently resolved by SDS PAGE. The gel was washed for ten minutes in water and imaged with Typhoon FLA 9500 (*GE Healthcare*) prior to Coomassie staining. The image was adjusted in Quantity One 1-D Analysis Software 4.5.1 (*BioRad*) and the grays were converted to red with ImageJ 1.51g (*National Institutes of Health*, USA).

4 References

1. Du, J.; Jiang, H.; Lin, H. *Biochemistry* **2009,** *48* (13), 2878-2890.

2. Wang, Y.; Rösner, D.; Grzywa, M.; Marx, A. *Angew. Chem. Int. Ed.* **2014,** *53* (31), 8159-8162.

3. Wallrodt, S.; Buntz, A.; Wang, Y.; Zumbusch, A.; Marx, A. *Angew. Chem. Int. Ed.* **2016,** *55* (27), 7660-7664.

4. Buntz, A.; Wallrodt, S.; Gwosch, E.; Schmalz, M.; Beneke, S.; Ferrando-May, E.; Marx, A.; Zumbusch, A. *Angew. Chem. Int. Ed.* **2016,** *55* (37), 11256-11260.
